# Sex-specific Differences in Infranodal Conduction Properties in Patients Undergoing Transcatheter Aortic Valve Replacement

**DOI:** 10.1007/s12265-023-10366-w

**Published:** 2023-03-08

**Authors:** Teodor Serban, Sven Knecht, Diego Mannhart, Thomas Nestelberger, Christoph Kaiser, Gregor Leibundgut, Maurice Antoine Bischof, Christian Sticherling, Michael Kühne, Patrick Badertscher

**Affiliations:** 1 Department of Cardiology, University Hospital Basel, Petersgraben 4, 4031 Basel, Switzerland; 2 Cardiovascular Research Institute Basel, University Hospital Basel, Petersgraben 4, 4031 Basel, Switzerland

**Keywords:** Arrhythmia, His Bundle, AV Node

## Abstract

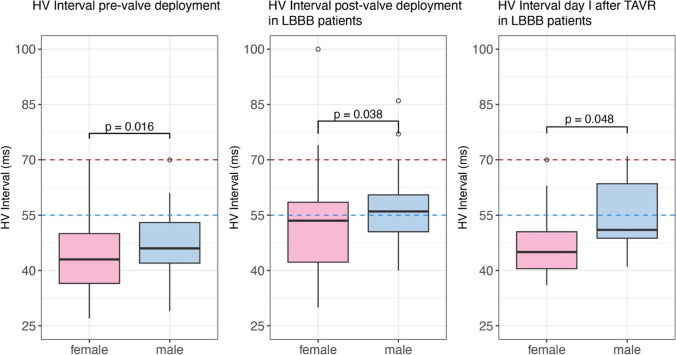

Electrophysiological (EP) testing for the assessment of atrioventricular (AV) conduction behaviour was proposed for risk stratification of patients with left bundle branch block (LBBB) after transcatheter aortic valve replacement (TAVR) [1]. Since sex-specific periprocedural data regarding infranodal conduction properties are lacking, we set out to compare the HV interval in men and women undergoing TAVR.

We retrospectively analyzed consecutive patients undergoing TAVR between August 2014 and June 2021 at the University Hospital of Basel. EP testing was performed pre- and immediately post-valve deployment and in the case of LBBB the following day. A surface 12-lead electrocardiographic (ECG) was available before and the day after the procedure in all patients to allow evaluation according to current guidelines [2]: EP testing is indicated in patients with new-onset LBBB and QRS-width ≥150ms or PR-interval ≥240ms and in patients with preexisting LBBB and an increase of the PR-interval or QRS-width by ≥ 20ms after TAVR.

127 patients were included in the study. The mean age was 81±7 years, 46% were female and the most frequently used valve types were the Evolut R or Evolut R Pro (41%) and Symetis ACURATE TA (39%). Median HV interval pre-valve deployment was 43[37-50] ms in women and 46[42-53] ms in men (*p*=0.016, Fig. 1), respectively and 13 patients (10%) had preexisting LBBB (six women and seven men).

**Fig. 1:**
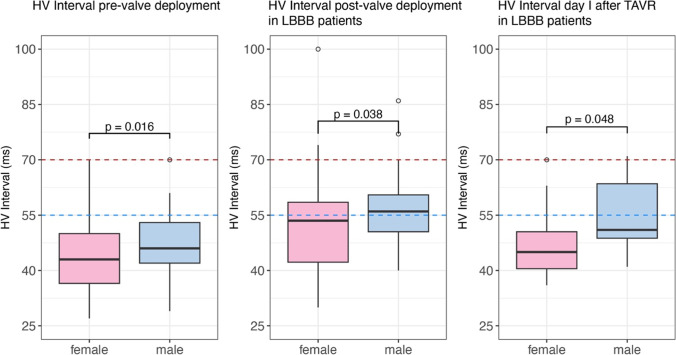
Boxplots showing the HV intervals pre-valve deployment (left), post-valve deployment (middle) and on day I after TAVR in LBBB patients for women (pink) and men (blue). The blue dotted line indicates 55 ms. The red dotted line indicates 70 ms

73 patients (57%) demonstrated LBBB post-valve deployment (67% women vs 49% men, *p*=0.06). The median HV interval in these was 54[42–59] ms in women and 56[51–61] ms in men (*p*=0.038). As such, a HV interval >55 ms or ≥70 ms was present in 31% and 8% of women vs 59% and 15% of men, respectively (*p*=0.049 and *p*=0.64). The median increase in HV interval pre- and post-valve deployment in patients with new LBBB was 7[2–11] ms in women and 11[3-18] ms in men (*p*=0.27). Nine patients (7.1%) underwent peri-procedural PM implantation due to third-degree AV block (5.2% women and 8.7% men, *p*=0.64).

Among the 73 patients with LBBB post-valve deployment, LBBB resolved the day following TAVR in 44 patients (61% women and 39% men, *p*=0.027). The 29 patients (41% female and 59% men) that demonstrated persistent LBBB the day after TAVR were invasively studied a third time. The median HV interval the day following TAVR in patients with LBBB was 45[41–49] ms in women and 50[48–62] ms in men (*p*=0.048), respectively and a HV interval >55 ms or ≥70 ms was present the day following TAVR in seven (24%) and three patients (10%). Two women (29%) and five men (71%) had HV intervals >55ms (*p*=0.75). One woman (33%) and two men (67%) had HV intervals ≥70ms (*p*=0.99). In these 29 patients with persistent LBBB the day after TAVR, EP testing was indicated according to current ESC guidelines criteria in 25 of 29 patients (86%). None of the four women without indication were found to have abnormal HV intervals.

To the best of our knowledge, this is the largest cohort to date to assess sex-specific differences in HV conduction in patients undergoing TAVR in the context of the current pacemaker guidelines [2]. We report several findings: 1) At baseline women have significantly shorter HV intervals than men. 2) While women more often develop LBBB post-valve deployment than men, the HV interval remains significantly shorter in women post-valve deployment. A prolonged HV interval of ≥55 ms was significantly less frequent in women than men post-valve deployment. 3) In 60% of patients with LBBB post-valve deployment, LBBB resolves the following day, significantly more often in females. The HV interval remains significantly shorter in women than men in patients with LBBB the day following TAVR.

Differences in electrophysiological characteristics of the AV conduction system between men and women have been described previously, outside of the TAVR population [3]. After TAVR, lesions of the conduction system in form of QRS duration prolongation, new LBBB or high-degree AV blocks requiring PM are common [1, 2, 4]. In the current study, we demonstrated that women have shorter HV intervals before TAVR and HV prolongation is less frequently noted after TAVR. Previous studies identified that women require less often PM after TAVR [4] and are more likely to recover the conduction disturbances during FU (seen as a reduction in ventricular pacing burden) provided the QRS duration improves and/or the LBBB resolves [5]. Our findings are hypothesis-generating, warranting further studies assessing the role of sex-specific cut-offs of the HV interval in women undergoing TAVR.

Limitations of our study include the retrospective design, low sample size and measurements of the HV interval the day following TAVR. The current ESC guidelines [2] recommend testing ≥3 days after TAVR.

In conclusion, women have shorter HV intervals pre- and post-valve-deployment as well as the day following TAVR compared to men. Further studies evaluating sex-specific cut-offs for the HV interval are warranted.
